# Effects of Agricultural Intensification on Mediterranean Diets: A Narrative Review

**DOI:** 10.3390/foods12203779

**Published:** 2023-10-14

**Authors:** Gultekin Hasanaliyeva, Enas Khalid Sufar, Juan Wang, Leonidas Rempelos, Nikolaos Volakakis, Per Ole Iversen, Carlo Leifert

**Affiliations:** 1 School of Animal, Rural and Environmental Sciences, Brackenhurst Campus, Nottingham Trent University, Nottinghamshire NG25 0QF, UK; 2 Nafferton Ecological Farming Group, Newcastle University, Newcastle upon Tyne NE1 7RU, UK; enasalkaisi@gmail.com (E.K.S.);; 3 Department of Clinical Nutrition, School of Medicine, Shanghai Jiao Tong University, Shanghai 200025, China; 4 Lincoln Institute for Agri-Food Technology, University of Lincoln, Lincoln LN2 2LG, UK; 5 Geokomi Plc, P.O. Box 21, GR70200 Sivas Festos, Greece; 6 Department of Nutrition, IMB, University of Oslo, 0317 Oslo, Norway; 7 Department of Haematology, Oslo University Hospital, 0424 Oslo, Norway; 8 SCU Plant Science, Southern Cross University, Military Rd., Lismore, NSW 2480, Australia

**Keywords:** Mediterranean diet, health impacts, phenolics/antioxidants, omega-3 fatty acids, Fe, I, Se, Zn, Cd, pesticides

## Abstract

Introduction: Mediterranean diets (MedDiets) are linked to substantial health benefits. However, there is also growing evidence that the intensification of food production over the last 60 years has resulted in nutritionally relevant changes in the composition of foods that may augment the health benefits of MedDiets. Objective: To synthesize, summarize, and critically evaluate the currently available evidence for changes in food composition resulting from agricultural intensification practices and their potential impact on the health benefits of MedDiets. Methods: We summarized/synthesized information from (i) systematic literature reviews/meta-analyses and more recently published articles on composition differences between conventional and organic foods, (ii) desk studies which compared food composition data from before and after agricultural intensification, (iii) recent retail and farm surveys and/or factorial field experiments that identified specific agronomic practices responsible for nutritionally relevant changes in food composition, and (iv) a recent systematic literature review and a small number of subsequently published observational and dietary intervention studies that investigated the potential health impacts of changes in food composition resulting from agricultural intensification. Results and Discussion: There has been growing evidence that the intensification of food production has resulted in (i) lower concentrations of nutritionally desirable compounds (e.g., phenolics, certain vitamins, mineral micronutrients including Se, Zn, and omega-3 fatty acids, α-tocopherol) and/or (ii) higher concentrations of nutritionally undesirable or toxic compounds (pesticide residues, cadmium, omega-6 fatty acids) in many of the foods (including wholegrain cereals, fruit and vegetables, olive oil, dairy products and meat from small ruminants, and fish) that are thought to contribute to the health benefits associated with MedDiets. The evidence for negative health impacts of consuming foods from intensified conventional production systems has also increased but is still limited and based primarily on evidence from observational studies. Limitations and gaps in the current evidence base are discussed. **Conclusions:** There is now substantial evidence that the intensification of agricultural food production has resulted in a decline in the nutritional quality of many of the foods that are recognized to contribute to the positive health impacts associated with adhering to traditional MedDiets. Further research is needed to quantify to what extent this decline augments the positive health impacts of adhering to a traditional MedDiet.

## 1. Introduction

Over the last 60 years, the intensification of agricultural practices (often referred to as the “green revolution”) has resulted in a substantial increase in not only global food production but also in the productivity of food production systems when assessed as (i) crop yields/unit area or (ii) meat, egg, or milk yield per livestock unit [1,2,3]. For example, globally, cereal production and cereal yields/ha were estimated to have doubled between the 1960s and the early 2000s [1] but have stagnated since then in many regions [4].

It is well documented that the increase in agricultural production/productivity was primarily due to (i) advances in crop and livestock breeding/selection, (ii) more widespread use and increased input levels of mineral N, P, and K fertilizers, crop protection products and irrigation water in crop production, and (iii) the increased use of indoor production, cereal and legume grain-based concentrate feeds, and veterinary medicines in livestock production, although (iv) a substantial expansion of the land area used for agricultural production has also contributed to the increase in global food production [1,2,3,4,5,6,7,8,9].

However, there is now also mounting evidence that many of the innovations introduced during the “green revolution” had negative impacts on the nutritional quality and safety of crop and livestock products used for human consumption, including many foods that are important components of the Mediterranean diet [4,6,7,8,9,10,11,12,13,14,15,16,17,18,19,20,21,22,23,24,25,26,27,28,29,30,31,32,33,34,35,36]. Different types of studies produced evidence for this, including investigations that compared the following:

- Foods from conventional production systems (which are based on innovations, inputs, and management practices developed/introduced during the green revolution) with foods from organic and/or traditional, extensive production systems which use (i) older and/or more resistant/robust crop and livestock genotypes and/or (ii) omit or restrict the use of many of the inputs and management practices (e.g., monoculture/short crop rotations; long indoor production periods for livestock) widely used in conventional systems [4,23,24,25,26,27,28,29,30,31,32,33];
- Food composition data from before and after agricultural intensification [10,11,12,13];
- The composition of foods from crop varieties or livestock breeds developed before and during the period of agricultural intensification [4,14,15,16,17,18,19,20];
- The composition of food crops produced with and without mineral fertilizers and/or pesticides introduced into crop production during intensification [4,5,6,7,8,9,18,19,20,21,22];
- The effects of organic and conventional food consumption on nutritional markers in animal and human dietary intervention studies [29,30,34,35,36].

Traditional Mediterranean diets (MedDiets) have been described as the “*gold standard in preventive medicine*” because they were shown to result in a lower prevalence of mortality from all causes and specifically a lower incidence of certain types of cancer, cardiovascular diseases, type 2 diabetes, neurodegenerative diseases, and overweight/obesity and associated comorbidities such as metabolic syndrome and kidney failure [34,35,37,38,39,40,41,42,43,44,45,46,47,48].

It is important to note that the evidence for the positive effects of MedDiets is based on both observational studies and randomized controlled dietary intervention trials (RCTs) and that the type and strength of evidence differs between the diseases/disorders listed above [34,35,37,38,39,40,41,42,43,44,45,46,47,48]. For example, there is strong evidence from RCTs for cardiovascular diseases, while the evidence for other diseases is based more on observational studies [34,35,37,38,39,40,41,42,43,44,45,46,47,48].

Traditional MedDiets are based on high levels of wholegrains, fruit, and vegetables but low red meat consumption, which is in line with current EU recommendations [5]. Apart from increased wholegrain, fruit, and vegetable consumption, the health benefits of the MedDiets have been linked with a high intake of fish, moderate wine consumption, and the use of olive oil [35,37,38]. It is also interesting to note that compared to Northern European diets, a larger proportion of red meat and dairy products consumed in a MedDiet is from small ruminants (goats, sheep), although there is limited information on potential health benefits [38].

**Figure 1:**
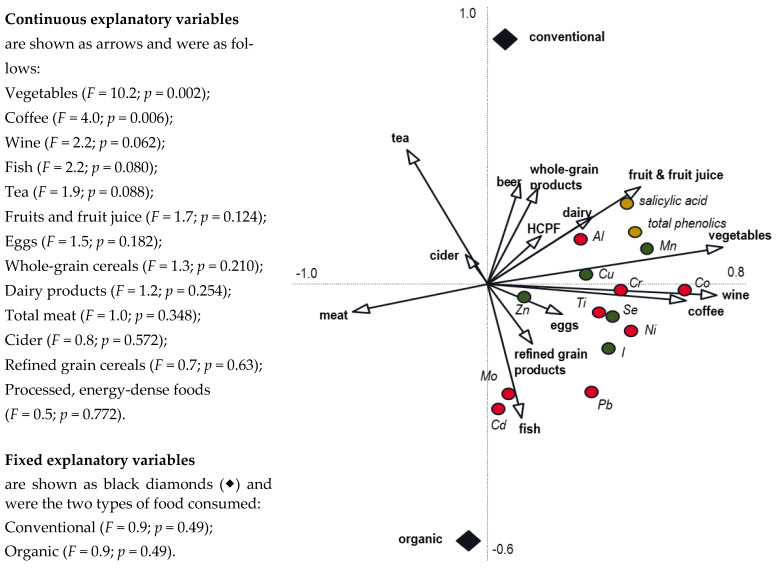
Biplot derived from the redundancy analysis showing the relationship between food type (organic vs. conventional) and diet component explanatory variables and urinary excretion of (a) salicylic acid (SA) and total phenolics (TP) as yellow circles, (b) mineral micronutrients (Cu, Fe, I, Mn, Se, Zn) as green circles, and (c) other metals (Al, Cd, Co, Cr, Mo, Ni, Pb, Ti) as red circles. Explanatory variables account for 33.6% of variation; the horizontal axis 1 explains 19% and the vertical axis 2 a further 6% of variation. Reproduced with permission from Rempelos et al., 2022 [35].

The health benefits were linked to high intakes of fibre, phytochemicals (e.g., polyphenols and carotenoids), and minerals (e.g., Cu, I, Zn, and Se) with antioxidant and/or anti-inflammatory activity and nutritionally desirable fatty acids (e.g., monounsaturated and very-long-chain polyunsaturated *n*-3 fatty acids—PUFAs) with MedDiets [35,36,37,38,39,40,41,42,43,44,45,46,47,48,49,50]. For example, we reported in a recent dietary intervention study that changing from habitual Western diets to a defined traditional MedDiet for 2 weeks increased (i) urinary excretion of total phenolics and salicylic acid by 46% and 45%, respectively, (ii) urinary excretion of the mineral micronutrients Co, Cr, I, Mn, and Se by 211%,22%, 70%, 102%, and 35%, respectively, and (ii) plasma Se concentrations by 14% [35]. Redundancy analyses of data from the same study [35] identified fruit, vegetable, and wine consumption as positive drivers for urinary excretion of total phenolics, salicylic acid, Co, Cr, Cu, I, Mn, and Se and meat consumption as a negative driver for the same list of micronutrients (Figure 1).

Similarly, a recent systematic review of observational studies and randomized controlled trials described that adherence to a MedDiet resulted in a significant increase in tissue *n*-3 PUFA levels in all observational and two-thirds of randomized controlled trials [50].

The underlying hypothesis for this narrative review was that agricultural intensification has significantly reduced the (i) nutritional quality of foods consumed as part of a MedDiet and (ii) health benefits of adhering to a MedDiet.

The objective of this review was therefore to synthesize, summarize, and critically evaluate the currently available evidence for changes in food composition resulting from agricultural intensification practices and their potential impact on the health benefits of MedDiets. The available information on the effects of agricultural intensification on the composition of (i) cereals, (ii) fruit and vegetables, (iii) meat and dairy products, and (iv) fish is described in Section 3.1, Section 3.2, Section 3.3 and Section 3.4 below. The limitations of the currently available evidence and the main conclusions are described in Section 3.5 and Section 4, respectively. The potential to overcome the potential negative impacts of intensification by changing to organic food consumption is also discussed.

## 2. Methods

The backbone of this narrative review is a summary/synthesis of results from (i) previously published systematic literature reviews/meta-analyses that compared the composition of conventional foods with organic products that are produced without most of the inputs and practices introduced during the period of agricultural intensification [6,23,24,25]. The meta-analyses in these studies were based on data from 343 studies on crops [24], 102 studies on fruit and vegetables [23], 196 studies on bovine milk [23], and 67 studies on meat from different livestock species [25].

Detailed descriptions of the literature search strategies and meta-analysis protocols used have previously been published [6,23,24,25]. Evidence from these systematic reviews was supplemented with information from a small number of desk studies which compared crop composition data from before and after agricultural intensification [10,11,12,13] and information from more recently published studies that (i) compared the composition of foods from intensive conventional and organic or low-input production systems and/or (ii) addressed gaps in the evidence base (e.g., data on Se concentration differences between wheat species and production systems; data on composition differences in milk from extensive and semi-intensive Mediterranean small ruminant production systems) (see Section 3 for lists of articles that contributed to the evidence base). Additional information and y articles were identified by the authors using similar literature search strategies to those described in the four systematic reviews [6,23,24,25].

The description of evidence for the effects of specific intensification practices on food quality was based on summarizing information obtained from two qualitative literature reviews [4,6] and/or more recent retail and farm surveys and/or factorial field experiments that identified specific agronomic practices responsible for nutritionally relevant composition differences between foods from conventional or intensive and organic or low-input production systems (see Section 3 for lists of articles that contributed to the evidence base).

The description of evidence for the potential impacts of agricultural intensification on human health was based on summarizing evidence from one recent systematic literature review [36] and a small number of subsequently published observational and dietary intervention studies that investigated potential health impacts of changes in food composition resulting from agricultural intensification (see Section 3 for lists of articles that contributed to the evidence base). The search strategy and methodology used in the systematic literature review is described in detail in the original article [36]. Subsequently published articles were identified using similar search strategies to those described in the original systematic review [36] (see Section 3 for lists of articles that contributed to the evidence base).

## 3. Results and Discussion

### 3.1. Effects of Intensification on the Nutritional Composition of Cereals and Grain Legumes

Wholegrain cereals (e.g., wheat, barley, oats, rice) and grain legumes (e.g., beans, peas, chickpeas, carob) and processed foods made from them (bread, pasta, couscous, fava, humus, etc.) are important components of traditional MedDiets [37,38,39,40,41,42,43,44,45,46,47,48,49,51,52]. The health benefits of wholegrain consumption have been linked to an increased intake of fibre, (poly)-phenolics, antioxidants, and mineral micronutrients (e.g., Ca, Zn, Se) [51,52,53,54,55].

While the effect of cereal breeding/selection and changes in agronomic practices on the nutritional composition of cereals has been studied extensively [4,14,15,17,18,19,20,21,22,23,24,28,29,30,31,32,33,34,35], there is very limited information for grain legumes [13]. Also, most studies that investigated composition differences between organic and conventional grain crops published over the last 10 years focused on cereals [4,28]. This section therefore primarily summarizes information from studies on cereals and, in particular, wheat.

For cereals, changes in (i) crop genetics, (ii) agronomic practices (fertilization, crop protection), and (iii) grain processing/post-harvest quality assurance practices were all shown to have affected the nutritional composition of grain [4,14,15,17,18,19,20,21,22,23,24,28,29,30,31,32,33,34,35], and the impacts of these three factors are therefore described separately in Section 3.1.1, Section 3.1.2 and Section 3.1.3 below.

#### 3.1.1. Wheat Genetics

One of the main breeding approaches used to increase the harvest index (the proportion of grain in the total biomass) and grain yields in cereals was the introduction of semi-dwarfing genes. This not only increased grain yields but also reduced straw length and thereby the risk of lodging, which had increased when farmers started to use high mineral N fertilizer inputs, as recommended during the “green revolution” [4,28]. Modern varieties of cereals (in particular wheat and rice) therefore have (i) shorter stems/straw and (ii) a higher maximum yield potential with high mineral fertilizer and pesticide input regimes typical for intensive conventional farming systems (see Section 3.1.2 Agronomic Practices).

A range of studies compared the composition of contrasting hexaploid wheat (*Triticum aestivum*) varieties and reported (i) lower concentrations of protein and nutritionally desirable mineral micronutrients (e.g., Cu, Fe, Se, and/or Zn) and/or phytochemicals (e.g., phenolics) and/or (ii) higher concentrations of carbohydrates and the toxic metal cadmium (Cd) in modern short-straw varieties, when compared with older (released pre-1960s), traditional, and/or longer-straw/stem varieties [14,15,16,17,18,20,28,56,57,58]. The magnitude of the reduction in modern wheat varieties differed between minerals, and the most notable reductions were reported for Mg, Fe, Se, and Zn [14,15].

Considerable variation in mineral nutrient concentrations between wheat species and varieties was reported in an extensive field experimental study by Zhao et al. [58], who compared 150 lines of common wheat (*T. aestivum* var. *aestivum*) with 10 lines of durum (*T. turgidum* var. *durum*), 5 lines of spelt (*T. aestivum* var. *spelta*), 5 lines of einkorn (*T. monococcum* var. *monococcum*), and 5 lines of emmer (*T. turgidum* var. *dicoccum*) wheat species. They found that the spelt, einkorn, and emmer lines had higher Se concentrations compared with common and durum wheats. When comparing the common wheat lines, they found that (i) grain Zn but not Fe concentrations correlated negatively with grain yield and (ii) that there was a “*decreasing trend in grain Zn concentration with the date of variety release, suggesting that genetic improvement in yield has resulted in a dilution of Zn concentration in grain*”, which is consistent with results from other studies [14,15,17,18].

Also, the results from a recent retail flour survey, in which samples of all brands of common wheat (*n* = 112) and spelt (*n* = 55) wheat available in German and UK supermarkets were analysed over two consecutive years, suggest that flour from currently used spelt wheat varieties (*T. spelta*: a hulled wheat species which was an important staple food in Europe between the Bronze age and medieval times but is now a minor cereal) has significantly higher mineral micronutrient (Cu, Mg, Zn) and phenolic concentrations and higher antioxidant activity (TEAC) compared with flour produced from modern common wheat (*T. aestivum*) varieties [32,33]. Compared with common wheat, there has been very limited breeding/selection effort for spelt wheat over the last 60 years, and the currently used spelt wheat varieties have a lower maximum yield potential and longer straw/stems but are more robust and require lower mineral NPK fertilizer and pesticide inputs to achieve their yield potential [19,20,32,33].

It is interesting to note that conventionally produced wholegrain spelt flour also had lower pesticide residues compared with common wheat wholegrain flour in the same retail flour survey [33]. In addition to differences in agronomic practices used for spelt and common wheat (see Section 3.1.2 below), this is thought to be partially due to the hull protecting the grain against becoming contaminated by pesticides applied late in the growing season [28,33].

Except for the positive correlations of grain micronutrient concentrations and negative correlation of grain cadmium concentrations with straw length, there is limited information about the potential morphological and physiological traits that are responsible for the differences in nutritional composition between old/traditional and modern wheat species or varieties. One potential explanation is that the higher yields of modern varieties result in a “dilution effect”, although this does not explain the higher Cd concentrations in grain from modern wheat varieties.

It has been suggested that the introduction of semi-dwarfing genes (which affect plant growth regulator concentrations in plants) has not only reduced stem length but also resulted in changes to root morphology, physiology, and distribution pattern in soil and that this may have had knock-on effects on mineral micronutrient uptake and/or redistribution [28,57]. There is, to our knowledge, no strong experimental evidence to support this hypothesis. However, the results of a mineral analysis of archived wheat grain and soil samples from the Broadbalk Experiment at Rothamsted Research Station in the UK provides some circumstantial evidence [59]. This unique long-term experiment allowed trends in grain mineral composition to be assessed in relation to soil mineral levels, wheat cultivar, yield, and harvest index [59]. The concentrations of Cu, Fe, Mg, and Zn were found to remain stable between 1845 and the mid 1960s but decreased thereafter, which coincided with the introduction of semi-dwarf high-yielding cultivars in the UK from the mid-1960s onwards. Decreasing grain concentrations were recorded in crops grown (i) without fertilizer inputs, (ii) mineral fertilizers, or (iii) manure inputs, and soil concentrations of the same mineral micronutrients either increased or remained stable between the mid-1960s and the early 2000s. This further supports the hypothesis that (i) changes in crop physiology associated with the introduction of semi-dwarfing genes and/or (ii) a dilution effect associated with increasing grain yields were the main driver for the decrease in Cu, Fe, Mg, and Zn observed during the period of agricultural intensification. It is important to note that multiple regression analysis identified both increasing yield and harvest index as highly significant factors that explained the decrease in grain mineral concentration [59].

The strong selection for yield against the background of high mineral fertilizer (and in particular P) inputs may have also co-selected against the capacity of modern varieties to establish mycorrhizal associations, which are known to facilitate the uptake of P and nutritionally relevant mineral micronutrients such as Zn [60,61,62,63]. There has been limited research into the impact of wheat breeding on mycorrhizal competence and associated micronutrient uptake capacity in modern compared with older/traditional varieties. However, it is interesting to note that a study which compared the relative dependence on mycorrhizas of modern wheat varieties, landraces, and ancestral wheat genotypes reported a trend for greater reliance on the symbiosis for yield in older cultivated wheat varieties [60]. Also, phytohormone concentrations in cereals, which were augmented by the introduction of semi-dwarfing genes, have also been identified as important regulators of AM symbiosis [61]. Furthermore, there is evidence for complex interactions between (i) wheat genetics (old versus modern varieties), (ii) agronomic practices (e.g., the use of water-soluble P fertilizers, which are known to augment mycorrhizal development), and (iii) the mycorrhizal populations developing in soils under different management regimes (e.g., organic versus conventional) [62].

Different to other mineral micronutrients (e.g., Cu, Fe, Zn), plant roots can actively take up Se as selenate (SeO^2−^, which is chemically similar to sulphate SO_4_^−2^) via high-affinity sulphate transporters in the root cortex, root tip, and lateral roots. The large differences in Se concentrations found between modern and older, long-straw wheat varieties reported by Murphy et al. [14] in the USA, where soils have relatively high Se concentrations, may therefore also have been due to breeding/selection having affected the density, activity, or distribution of sulphate transporters in wheat roots [14]. It is interesting to note that in the Broadbalk Experiment, the trends for Se grain concentrations over time differed from those observed for other mineral micronutrients [64]. This experiment was carried out in the UK, where soil Se concentrations are known to be low, and showed that (i) the introduction of semi-dwarf, high-yielding wheat varieties did not coincide with a decrease in grain Se levels, (ii) soil Se levels increased over time, (iii) grain Se concentrations were lower in non-fertilized control plots and not significantly affected by fertilizer type, and (iv) grain Se was significantly negatively correlated with SO_2_ emissions in the UK [59]. In contrast, results from long-term factorial field experiments carried out at Newcastle University’s Nafferton Experimental Farm in the UK [57] suggest that longer-straw spelt varieties had higher grain Se concentrations than modern short-straw common wheat varieties and that the use of mineral NPK resulted in lower grain Se concentrations compared with manure applied at the same N input level (see also Section 3.1.2 below). In these unique experiments, (i) rotation design, (ii) fertilization regimes, (iii) crop protection protocols, and/or (iv) crop species/variety were used as factors and therefore allowed both main effects and interactions between these agronomic parameters to be identified and quantified (see Rempelos et al. [27,56,65] and Daud et al. [57] for detailed descriptions of the experimental designs).

In contrast to mineral micronutrients, concentrations of the toxic metal Cd were reported to be significantly higher in modern compared with older wheat cultivars when grown without fungicide applications [18]. The underlying mechanisms are unclear, although it should be noted that one reduced height gene (Rh8) has been associated with grain Cd [18]. However, Cd is known to be a complex trait and was also reported not to be clearly associated with straw/stem length [18,66].

There is also some evidence that breeding and selection for high yields against the background of conventional agronomic protocols (with high mineral NPK and pesticide inputs) has co-selected for a greater need for the application of synthetic chemical pesticides in cereal production [4,28,56]. For example, there is evidence that the short-straw modern wheat varieties are (i) less competitive against weeds and (ii) more susceptible to *Fusarium* grain infection (and associated mycotoxin contamination) from fungal inoculum on crop residues on the soil surface [4,28,31]. Results from the long-term Nafferton Factorial Systems Comparison (NFSC) trials and other factorial field experiments carried out between 2000 and 2017 at Nafferton Farm in the UK also provided evidence that organic common wheat breeding programmes with selection under low-input organic background conditions may have generated varieties with (i) longer straw/stems, (ii) greater resistance to biotrophic diseases such as powdery mildew and rust, and (iii) higher leaf phenolic concentrations [4,28,56].

It is important to note that positive correlations between straw length and higher mineral micronutrient concentrations were reported in all studies with common wheat, while one factorial field experiment (with variety, irrigation, and fertilizer type as factors) carried out in Crete, Greece, which compared two traditional long-straw spelt varieties with one modern short-straw spelt variety, reported significantly higher phenolic concentrations in the short-straw variety [20]. This may be explained by the differences in agronomic protocols used between common and spelt wheat and differences in genetic × agronomy interactions. Specifically, spelt wheat is produced with substantially lower nitrogen and pesticide inputs (both of which have been shown to affect phenolic concentrations in crops) compared with common wheat (see Section 3.1.2 for a description of the effects of N inputs and crop protection on grain phenolic concentrations). Also, many modern short-straw spelt varieties, including the one used in this study, were developed from *T. aestivum* × *T. spelta* crosses [20], while modern common wheat varieties were developed through the introduction of semi-dwarfing genes and selection for high yields with high mineral NPK fertilizer input regimes.

Similar studies are not available for other important wheat species (e.g., *T. durum*) and other cereals (barley, oats, rice) which are consumed as part of traditional MedDiets.

#### 3.1.2. Agronomic Practices

The evidence for changes in agronomic practices having affected the nutritional composition of cereals comes primarily from studies which (i) investigated the effect of specific inputs/practices (e.g., mineral NPK fertilizers, synthetic chemical pesticides, short rotations/cereal monoculture, minimum/no tillage) introduced during the green revolution and (ii) compared the nutritional composition of conventional and organic cereal grains/products [4]. Organic cereal production resembles in many aspects the type of agronomic protocols used prior to the green revolution because it uses (i) no synthetic chemical pesticides and mineral N, P, and KCl fertilizers, (ii) more diverse rotations which include legume crops, and in most organic cereal production protocols, (iii) mechanical weed control and traditional inversion ploughing-based tillage protocols and (iv) legume fertility-building crops, animal manures, and/or organic waste-based composts to maintain soil fertility [4].

Phenolic concentrations in the leaves and grain of common wheat (*T. aestivum*) were shown to (i) decrease with increasing mineral N fertilizer inputs and (ii) be lower with mineral N fertilizer when compared with cattle farmyard manure applied at the same total N input level [4,29,30,56,66]. Phenolics are part of the plants constitutive and inducible resistance response to disease attack, and it has been demonstrated that increasing N availability to plants significantly reduces the concentrations of phenolic compounds and resistance against biotrophic diseases in wheat and other crops in a dose-dependent manner [66,67,68,69,70]. However, results from the Nafferton Factorial Systems Comparison (NEFG) trials also showed that the use of conventional crop protection regimes also has a significant negative effect on phenolic levels in cereals but not field vegetables [see Section 3.2 below] and that there are significant interactions between crop protection and fertilization for phenolic concentrations in both wheat and barley [28,56].

**Figure 2:**
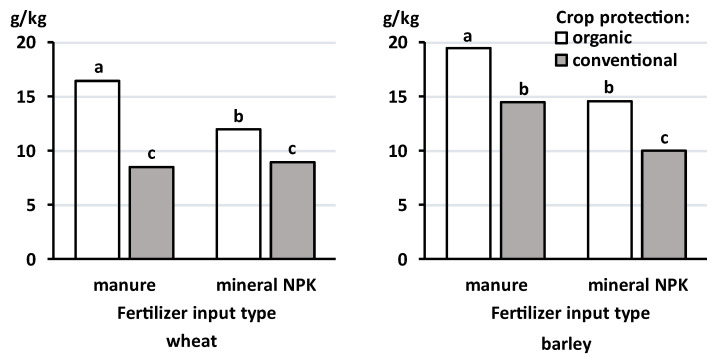
Effect of crop protection and fertilization regimes used in organic and conventional production systems on the total polyphenolic concentrations in compound feeds made from cereal grain (wheat or barley), potato, carrots, and onions grown in the NFSC trials. Data are from Średnicka-Tober et al. [29] and Baranski et al. [30]. Bars labelled with the same letter in the same graph are not significantly difference (*p* < 0.05).

Figure 2 shows the effects of fertilization and crop protection regimes used in organic and conventional crop production on the total polyphenolic concentrations in experimental animal feeds used in dietary intervention studies [29,30]. Feed was made from cereal (55% *w*/*w*), potato (10%), carrot (4%), and onion (1%) crops harvested from plots managed with contrasting management protocols during the NEFG trials [29,30]. The results indicate that phenolic concentrations were (i) generally lower when wheat was used as the cereal component in feed and (ii) ~90% higher in organic compared with conventional feed and (iii) that the trend for the interaction differed between wheat and barley (Figure 2). In this context, it is interesting to note that the plant growth regulator chlormequat is used in wheat but not barley (which is less susceptible to lodging than wheat) as part of the conventional crop protection regime [29,30].

Se concentrations in grain were recently shown to be significantly lower in spelt wheat crops fertilized with mineral NPK (NPK) or maize biogas digestate (MBD) compared with crops fertilized with composted cattle farmyard manure (FYM) applied at the same total N input level [57]. In contrast, yields were highest with MBD digestate, intermediate with NPK, and lowest with FYM as fertilizer. This and the finding of significantly higher Se concentrations in FYM- compared with MBD-fertilized crops suggests that the higher Se inputs with FYM compared with MBD and NPK (which contains virtually no Se) were the main driver for the higher Se concentrations in FYM-fertilized crops. Lower Se concentrations in NPK- compared with FYM-fertilized crops were also found in factorial field experiments with common wheat [57]. It is important to note that concentrate feeds supplemented with Se and other mineral micronutrients are routinely used in both conventional and organic cattle production in Europe, which explains the higher Se concentrations in FYM compared with MBD in this study [57]. It is also important to consider that mineral supplementation of animal feeds was not widely used before the 1960s and can be considered an innovation that was introduced as part of the green revolution. Similarly, the introduction of Se supplementation of mineral N fertilizers in Finland was successfully used to increase Se concentrations in soils, cereals, and other crops, and this is also clearly an innovation introduced as part of the agricultural intensification that improved dietary micronutrient intake [57,71].

Cd inadvertently accumulates in wheat grains, and wheat is recognized as a primary source of dietary Cd intake [18]. Cd concentrations in wheat grain and other cereals are well known to increase with increasing mineral P fertilizer inputs, and in intensive conventional cereal production systems/regions (e.g., rice/maize or wheat/maize double cropping rotations in China) which use very high mineral P fertilizer inputs (~600 kg P/ha), Cd concentrations in grain can be above the thresholds set by the WHO [4,18,22,65,72]. Also, long-term factorial field trials with common wheat have shown that NPK-fertilized common wheat crops have higher Cd and/or Ni concentrations compared with crops fertilized with FYM at the same total N input level [21]. In contrast, in spelt (which is produced with much lower mineral fertilizer inputs), no significant difference in Cd concentrations could be detected between manure and NPK applied at the same total N input level [20].

Pesticide residues in cereals and other grain crops have also increased since they were first made widely available and used by farmers in the 1960s [73,74,75,76]. However, data from regulatory pesticide monitoring in Europe [73,74,75,76] also show that the profile of pesticides used has changed over time, and total pesticide inputs to wheat and other cereals have decreased since the 2000s in some regions (e.g., the EU), although this decrease may have been due to older pesticides (e.g., S-fungicides and insecticides) being replaced by new pesticide products that have activity at much lower application rates [73,74,75,76].

Systematic reviews and meta-analyses of data from studies which compared the pesticide residues/profiles in organic and conventional cereals and other crops reported that conventional cereal grains/products (which are produced with crop protection protocols based on multiple pesticide applications) have substantially higher pesticide residue levels compared with organic cereal grains (which are produced without applications of synthetic chemical pesticide) [24,77]. Similar results were recently found in an extensive wheat flour survey conducted in the UK and Germany, which detected a larger number of different pesticide residues and higher pesticide residue concentrations in conventional compared with organic cereals [33].

It should be noted that the evidence for lower pesticide residues in organic compared to conventional food crops is now widely accepted, while there is still controversy about the evidence for other food quality, safety, and security benefits of organic food production and consumption [78,79]. In cereals, the use of (i) the plant growth regulator chlormequat (which is used to reduce stem length and thereby prevent the lodging of cereals) and (ii) applications of the herbicide glyphosate close to harvest to desiccate cereals is currently of particular concern. This is mainly because (i) these practices result in high residue levels of chlormequat, which is a very persistent chemical linked to endocrine-disrupting activity that has been banned for use in fruit production but is still permitted for use in wheat, and (ii) glyphosate has been classified as a probable carcinogen [33,34,74,75,76,80]. There is also evidence that applications of glyphosate as (a) a pre-emergence treatment in non-glyphosate-resistant soybean and (ii) during the growing season in glyphosate-resistant soybean crops impairs plant micronutrient uptake and thereby reduces concentrations of some micronutrients in soybean leaves and seed [81,82,83].

**Figure 3:**
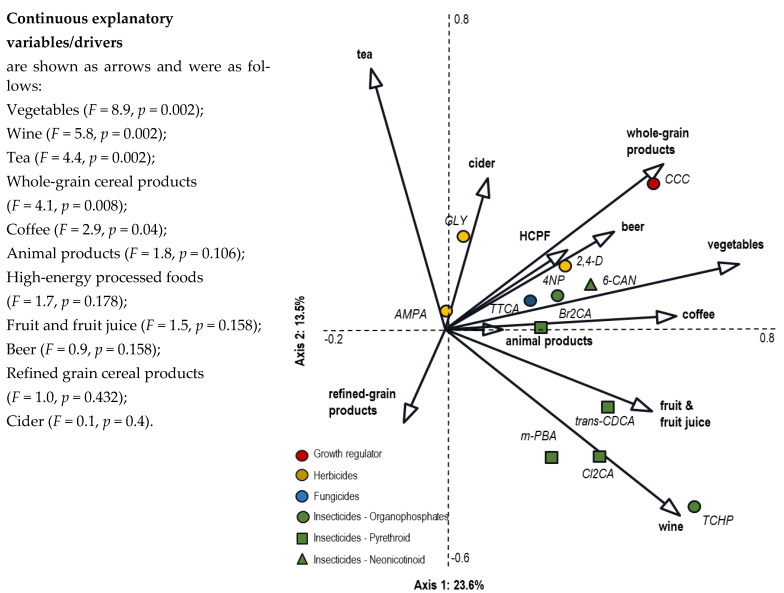
Biplot derived from the redundancy analysis of data from conventional food consumption before, during and after the intervention period only showing the relationship between diet component explanatory variables and urinary excretion of (a) growth regulator (CCC, chlormequat), (b) herbicide (GLY, glyphosate; AMPA, amino-methyl-phosphoric acid; 2,4-D, 2,4-dichlorophenoxyacetic acid), (c) dithiocarbamate and trihaloalkylmercaptoimide fungicide (TTCA, 2-thio thiazolidin-4-carboxylic acid), (d) pyrethroid insecticide (Cl2CA, cis-/trans-3-(2,2-dichlorovinyl)-2,2-dimethylcyclopropanecarboxylic acid; Br2CA, cis-3-(2,2-dibromovinyl)-2,2-dimethyl cyclopropane carboxylic acid; m-PBA, 3-phenoxybenzoic acid; t-CDCA; trans-chrysanthemumdicarboxylic acid), (e) organophosphate insecticide (4NP, 4-nitrophenol; TCHP, 3,5,6-trichloro-6-hydroxypyridine), and (f) neonicotinoid insecticide (6-CNA, 6-chloronicotinic acid) residues. Explanatory variables accounted for 40.1% of total variation; axis 1 explains 23.6% and axis 2 a further 13.5% of variation. Reproduced with permission from Rempelos et al., 2020 [34].

A range of studies have shown that cereal grains are an important source of dietary pesticide exposure [4,71,72,73,78,79]. Also, a recent dietary intervention study [34] demonstrated that the consumption of wholegrain cereal products (which includes beer) is closely associated with urinary excretion (a marker for total pesticide exposure) of (i) the plant growth regulator chlormequat, (b) the herbicides 2,4-D and glyphosate, and (iii) neonicotinoid and organophosphate insecticide metabolites (Figure 3). This suggests that conventional wholegrain cereal products are a major dietary source of these compounds.

*Fusarium* mycotoxin contamination is also affected by many of the agronomic practices introduced during the intensification of conventional cereal production over the last 60 years, and this has recently been reviewed in detail by Bernhoft et al. [84]. Briefly, there is evidence that high mineral N inputs, the use of chlormequat to shorten stems, increased stem density, the use of certain fungicides (e.g., strobilurins), no and minimum tillage, and cereal monoculture/non-diverse arable rotations (and, in particular, growing wheat after maize) increase the risk of *Fusarium* mycotoxin contamination in cereals [84]. It is important to note that mycotoxin levels in wheat grain in the 27 large well-designed farm surveys reviewed by Bernhoft et al. [84] were substantially higher than the concentrations found in both wholegrain and white wheat flour in the most recent retail survey conducted by Wang et al. [31]. This difference is thought to be primarily due to the introduction of quality assurance protocols by grain processors that involve mycotoxin testing of all batches of cereals destined for human consumption in order to comply with maximum contamination levels (MCLc) set by the EU [31,84]. It also, at least partially, explains why most farm surveys reviewed by Bernhoft et al. [84] found significantly higher concentrations of *Fusarium* mycotoxins in conventional cereal grain, while Wang et al. [31] found similar concentrations of both (i) *Fusarium* mycotoxins and (ii) ochratoxin A in organic and conventional wholegrain wheat flour. Since cereals with mycotoxin levels above the MCL for humans are widely used as animal feed, the effects of agricultural intensification on mycotoxin levels in cereals are therefore more likely to have an impact on livestock than human health.

It is important to point out that recent factorial experimental and survey-based studies with wheat have also identified significant (i) *genetics* × *agronomy*, (ii) *environment* × *genetics* × *agronomy*, and (iii) *genetics* × *agronomy* × *processing* interactions for a range of nutritionally relevant compounds (including phenolics, mineral micronutrients, pesticide residues, mycotoxins) [4,19,20,28,31,32,33,56,57,58,84,85]. While explaining the large variation and sometimes inconsistency of results from studies carried out in different counties, seasons, and pedoclimatic environments identified in systematic reviews/meta-analyses of comparative crop composition data [23,24], this also highlights the risk of bias when conclusions about the effects of intensification on food quality are based on evidence from individual or only a small number of studies/environments.

#### 3.1.3. Grain Processing and Post-Harvest Quality Assurance Protocols

In the past, a large proportion of cereal-based foods consumed as part of a MedDiet were made from wholegrain, which is widely recognized to have significant positive nutritional (higher fibre, antioxidant, and mineral micronutrient intake) and associated health impacts [32,51,52,53,54,55]. However, as in Northern and Central Europe and North America, wholegrain consumption in many Mediterranean regions has declined over the last 60 years and been replaced by products made from refined grains/flour [54,55].

Refining substantially reduces the concentrations of a range of mineral micronutrients (e.g., Ca, Mg, Fe, Cu, Zn), phenolics, and other antioxidants that are mainly located in the outer bran and germ layers of the cereal grain [32,53]. In contrast, Se and Cd are known to be more uniformly distributed across the grain and found in similar concentrations in (i) the bran, germ, and endosperm and (ii) wholegrain and refined cereal flour [18,32,53,57].

However, refining is also increasingly recognized to have some nutritional benefits, especially when used for conventionally produced cereal grains [51]. Most importantly, refining is known to reduce the concentrations of pesticides, especially non-systemic pesticides and those applied late in the growing season [33,63]. Also, a recent wheat flour survey reported that refined flour contained substantially lower concentrations of the mycotoxin T-2/HT-2, which is known to be produced by *Fusarium* species that colonize the outer surface of the grain which is removed during refining [31].

It is important to note that some of the negative aspects of refining are addressed by the grain processing and efficient quality assurance protocols used in developed countries (e.g., compulsory fortification of refined cereals with minerals such as Ca or Fe and testing of all cereal batches for mycotoxins) [31,32,51].

For cereals, the evidence base for agricultural intensification having resulted in a decline in the nutritional quality has grown substantially since the publication of the comprehensive systematic literature reviews/meta-analyses of composition differences between organic and conventional cereal crops in 2014 [24]. Specifically, new evidence from long-term factorial field experiments and retail surveys have increased our understanding of how and to what extent changes in agronomic practices, crop genetics, and cereal processing introduced over the last 60 years have affected the concentrations of (i) phenolics (the main group of phytochemicals contributing to antioxidant activity in crop plants), (ii) Se and Zn (two mineral micronutrients for which cereals are a major dietary source), (iii) pesticide residues, (iv) the toxic metal Cd, and (v) *Fusarium* mycotoxins (for which cereals are the main dietary source). Recent studies have also increased our understanding of reasons for the large between-study variation found in meta-analyses of comparative composition data [24].

A comprehensive, factorial retail survey of wheat flour also facilitated a more accurate estimate of dietary intakes of micronutrients, pesticides, and mycotoxins or organic and conventional wholegrain and refined cereals products made from wheat (the most important cereal used for human consumption in Western Europe and North America) [31,32,33]. This survey also identified, for the first time, an interaction between diet choice (wholegrain vs. refined flour products) and food type (organic vs. conventional) by showing the following:

- Diet choice (consumption of wholegrain instead of refined flour products) has a more substantial impact on the intake of compounds linked to positive health impacts (including phenolics, antioxidants, and the mineral micronutrients Zn, Fe, and Cu) compared with the agronomic protocols (organic versus conventional) used to produce wheat grain;
- Food type (organic versus conventional) had a more substantial impact on the intake of nutritionally undesirable pesticides and plant growth regulator residues in wheat grain;
- The consumption of organic wholegrain products facilitates higher intakes of the nutritionally desirable compounds (compared with conventional wholegrain) without increased exposure to pesticide residues.

In this context, it is important to consider that the evidence base for cereals other than wheat is still relatively small for most nutritionally relevant compounds. An exception is the now substantial evidence base available for *Fusarium* mycotoxin contamination, which was subject to several literature reviews (see Bernhoft et al. [84] for a summary review). They consistently concluded that (i) organic production results in similar or slightly lower *Fusarium* mycotoxin contamination in a range of cereal species compared with cereals from conventional production and (ii) that *Fusarium* mycotoxin concentrations in both organic and conventional cereals/cereal products used for human consumption are well below the MCL in both wholegrain and refined cereal grains/products.

### 3.2. Effects of Intensification on the Nutritional Composition of Fruit and Vegetables

A high level of consumption of fruit and vegetables and specific processed foods made from them (e.g., wine, olive oil) is an important component of traditional MedDiets and their associated health benefits [35,37,38,39,40,41,42,43,44,45,46,47,48,49]. Health benefits were linked to (i) high fibre, (poly)-phenolic, antioxidant, and mineral micronutrient intake with fruit and vegetables, (ii) increased antioxidant and total phenolic (and in red wine also anthocyanin) intake from moderate wine consumption, and (iii) increased intake of phenolics and the monounsaturated fatty acid oleic acid with olive oil [35,37,38,39,40,41,42,43,44,45,46,47,48,49,86,87,88,89,90,91,92,93,94,95,96].

Fruit and vegetables consumed as part of MedDiets belong to a wide range of plant families that differ substantially with respect to (i) genetics, morphology, physiology, life cycle (e.g., annual, biannual, perennial), and growth pattern, (ii) methods used for propagation, and (iii) the pedoclimatic conditions and agronomic management practices (e.g., irrigation, tillage, pruning, crop protection, and fertilization) required for optimum performance. It was therefore not surprising that systematic literature reviews/meta-analyses of composition differences between organic and conventional crops identified substantial variation between studies focused on different crop species [23,24]. Both meta-analyses were carried out by synthesizing data from different crop species and identified significantly higher concentrations of phenolics and other nutritionally desirable phytochemicals in organic crops [23,24]. The meta-analysis by Średnicka-Tober et al. [24] also analysed data on mineral micronutrients and pesticide residues. While differences for mineral micronutrients (including Zn and Ca) were relatively small, they reported (i) significantly higher contamination levels in conventional than in organic fruit and vegetables and also (ii) higher levels in conventional fruit than in conventional vegetables. However, the information available did not allow (i) composition differences in specific vegetable and fruit crop species to be estimated and (ii) effects of specific agronomic practices (rotation, fertilization, crop protection) used in organic and conventional farming on crop quality parameters to be identified [24].

The relative efforts to improve crop yields and other performance parameters via breeding/selection over the last 60 years and the use of new breeding strategies (e.g., open-pollinated versus hybrid) differed significantly between fruit and vegetable species [4]. Also, different to cereals, there are, to our knowledge, no scientifically sound studies for fruit and vegetable species which compare the nutritional composition of varieties/cultivars/hybrids developed before the 1960s with modern genotypes developed in the last 30–40 years. It is therefore virtually impossible to draw general conclusions on the impacts of crop breeding on the nutritional composition of fruit and vegetables.

However, similar to cereals, it is well known that there is substantial variation in nutritional composition between varieties/hybrids/genotypes in many fruit and vegetable species. This includes grapes and tomatoes, which are considered staples and significant drivers for phenolic, vitamin, mineral, and antioxidant intake and the associated health benefits of MedDiets [37,38,39,40,41,42,43,44,45,46,47,48,49,50,84,85,86,87,88,89,90,91,92,93,94].

For example, a recent review of results from seven farm-survey-based studies that compared antioxidant activity and total phenolic and total antioxidant concentrations in organic and conventionally produced table grapes identified substantially larger effects of grape type (black vs. red vs. white) and grape variety than production system or production region/country [87]. Specifically, in white grapes, total antioxidant activity varied between 3 and 60 µmol Trolox equivalents (TE)/g in fruit from different white grape varieties and between 20 and 160 µmol Trolox equivalents TE/g in grapes from different red or black varieties. Total phenolic concentrations varied between 126 and 3378 gallic acid equivalents/kg and anthocyanins between 49 and 851 cyanidin-3-O-glucoside equivalents/kg in fruit from different red or black varieties [87]. These results are consistent with a recent retail survey of table grapes that investigated the effect of grape type (black, red, green/white), variety, production region (Southern Africa versus Mediterranean), and production system (organic versus conventional) based on an analysis of 410 matched samples (organic and conventional grapes of the same grape type collected on the same date; *n* = 820) on phenolic concentrations over two years [87]. Specifically, this study reported that, compared to black grapes, (i) total phenolic concentrations were 8% and 38% lower in red and white grapes, respectively, and (ii) total anthocyanin concentrations 83% and 98% lower in red and white grapes, respectively [88].

Recent comparisons of tomato cultivars/hybrids also reported substantial variation in total carotenoid, ascorbic acid, flavonoid, and total phenolic content and antioxidant activity [95,96,97,98,99,100]. Bhandari et al. [96] also reported variation in specific carotenoids, with the highest level of variation detected for lycopene, followed by β-carotene and lutein. One trend observed across most studies was that small fruit (cherry) tomato cultivars tended to have higher antioxidant and phenolic concentrations and lower yields than larger-fruiting varieties/hybrids. A study by Nour et al. [99] also reported significant variation in Ca, Mg, K, Na, Fe, Mn, Cu, Zn, and B concentrations between tomato cultivars/hybrids. 

There are also a small number of field experiments which have compared high- and low-yielding varieties/hybrids of some fruit and vegetable species (e.g., of broccoli), and a recent review of these studies described that they consistently showed negative correlations between yield and concentrations of minerals and protein indicative of a “dilution effect” [91].

In the section on fruit and vegetables we have therefore focused on summarizing the results from (i) studies that compared food composition data from before and after agricultural intensification (Section 3.1.1) and (ii) field experiments focused on comparing the effect of contrasting agronomic practices (especially fertilization and crop protection) on the nutritional composition of fruit and vegetables (Section 3.2.2).

#### 3.2.1. Evidence for Historical Changes in Nutrient Composition

Several studies have investigated historical changes in the mineral nutrient composition of fruit, vegetables, and/or nuts by comparing published food composition data from the 1960s or before (pre-agricultural intensification) with composition data obtained between the 1980s and 2010s (post-agricultural intensification) [10,11,12,13,92]. Although there is considerable variation between (i) studies, (ii) countries in which samples were collected, and (iii) between fruit and vegetable species, most studies have reported similar trends. For example, Table 1 summarizes the results from three UK and one US studies [10,11,13] that analysed government nutritional composition survey data for Ca, Mg, Cu, Fe, K, and P from the UK and USA. Specifically, they reported (i) no change in P concentrations in both fruit and vegetables, (ii) significant reductions in K and Fe concentrations in fruit and/or vegetables, and (iii) significant declines in Mg and Cu in both fruit and vegetables over time in both the USA and UK (Table 1).

Similar results for Ca and Fe were also reported in another US study [92] that also compared concentrations of macronutrients (carbohydrate, fat, and protein) and selected nutritionally desirable phytochemicals in 43 garden crops (mainly vegetables). The study detected significant declines in protein, Ca, P, Fe, riboflavin, and ascorbic acid content but did not find a significant decline for five other nutrients (fat, carbohydrate, vitamin A, Thiamin, and Niacin) [92].

Since (i) increasing marketable yield has also been the dominant breeding target in most vegetable and fruit crops and (ii) variety trials have consistently shown correlations between yield and concentrations of minerals and protein, it is tempting to assume that the historical decline in some minerals and phytochemicals is primarily due to a “dilution effect” [91]. However, there is evidence that changes in agronomic practices have also contributed to the declines in mineral and phytochemical concentrations (see Section 3.2.2 below).

It is also important to consider that, different to cereals, breeding/selection in a number of fruit and vegetable crops has more recently also focused on improving certain nutritional quality parameters (e.g., phenolic and antioxidant content) [93,94]. For these species, the (i) decline in certain nutritionally desirable compounds and (ii) the negative correlations with yield may have already started to reverse, although this has not been documented yet.

#### 3.2.2. Agronomic Practices

Recent factorial field experiments and retail surveys have provided evidence that, similar to cereals, agricultural intensification (especially the use of high mineral NPK fertilizer and pesticide inputs to increase yields) can reduce the nutritional quality of fruit and vegetables [4,27,29,30,34,68,69,70,74,75,76,77,78,79,80,87,88,89]. There is also now some evidence that excessive irrigation can have negative effects on both crop yield and quality [101].

Phenolic concentrations were found to decrease with increasing mineral N inputs in a range of fruit (e.g., grapes, apple) and vegetable crops (e.g., tomato, zucchini, potato), and in several crop species, this coincided with a reduction in disease resistance [68,69,70]. Also, recent farm and retail surveys with grapes reported that, overall (across all grape varieties assessed), organically produced grapes had higher phenolic concentrations and antioxidant activity than conventionally produced grapes, although it is important to note that variety had a substantially larger effect on grape composition than agronomic protocols [87,88].

These findings are consistent with the results of several systematic reviews and meta-analyses, which found lower poly(phenolic) concentrations and antioxidant activity in fruit and vegetables from conventional production systems (which use pesticides for crop protection and rely nearly exclusively on mineral NPK fertilizers for crop nutrition) compared with produce from organic production (which is produced without synthetic chemical pesticides, mineral N, water soluble P, and KCl fertilizers) [23,24,26,79,102].

Results from the long-term Nafferton Factorial Systems Comparison (NFSC) trials at Newcastle University (Newcastle Upon Tyne, UK) suggest that the differences in phytochemical composition between organic and conventional vegetables are relatively small and primarily due to the contrasting fertilization regimes used in organic and conventional production (Table 2). Specifically, the NFSC trials showed that the use of mineral NPK fertilizer (NPK) results in higher N availability but significantly lower total phenolic levels in potato, cabbage, and lettuce, but not onions, compared with crops grown with cattle farmyard manure (FYM) applied at the same N input level [27]. It is important to consider that (i) only ~50% of total N applied with FYM is known to become plant through mineralization in the first growing season, (ii) crop yields were significantly lower with FYM compared with NPK in potato, cabbage, and lettuce but not onion crops, and (iii) that the use of NPK also resulted in significantly lower glucosinolate and carotenoid concentrations in cabbage, vitamin C concentrations in potato and cabbage, and vitamin B_9_ concentrations in potato and lettuce compared with NPK [27]. This may indicate that both the (i) down-regulation of phytochemical synthesis by the higher N availability from NPK and (ii) a dilution effect resulting from higher yields may have contributed to the lower phenolic, carotenoid, and vitamin concentrations in NPK-fertilized potato, cabbage, and lettuce [27].

In contrast, (i) the preceding crop (spring beans versus winter barley) only had a significant effect on the vitamin C content in cabbage (higher in crops grown after winter barley), and (ii) crop protections (pesticide-based conventional versus mechanical weed control and insect proof crop cover-based organic) only affected the glucosinolate and vitamin B_9_ content in cabbage (both lower with organic crop protection) [27]. The study concluded that these effects were most likely due to the higher N availability following a legume crop and a reduction in solar irradiation by crop covers reducing the synthesis of phytochemicals in organically produced cabbages [27].

Similar to cereals, recent studies [34,36] confirmed that (i) conventionally produced fruit and vegetables have substantially higher pesticide residues than organic produce, and (ii) contamination levels are higher in conventional fruit than in conventional vegetables.

However, based on the still very limited evidence available, it is still not possible to identify consistent overall trends for the effects of (i) production systems (e.g., organic vs. conventional) and (ii) specific agronomic parameters (e.g., fertilizer type/input levels, crop protection protocols, rotation, tillage) on mineral micronutrient concentrations in fruit and vegetable crops [4,26,27,78,79].

The consumption of olive oil is thought to be a particularly important driver for monosaturated fatty acid (oleic acid) intake and associated health benefits of the MedDiet [47,48,49,50]. Increasing olive oil/oleic acid consumption was linked to a reduction in plasma cholesterol, LDL cholesterol, and triglycerides, an improvement in immune function, and protection against atherosclerosis in mice and, potentially, anti-cancer properties [103,104,105]. Recent farm surveys and experimental studies in Greece that compared the performance of organic and conventional olive production systems reported no significant differences in nutritional and sensory composition parameters (including oleic acid and phenolic concentrations and acidity) between organic and conventionally produced table olives and olive oil, except for higher pesticide residues in conventional olive oil and fruit [89,106,107,108]. It is important to note that (i) olive fruit and oil yields were not significantly different and were numerically slightly (~10%) higher in organic production systems [89], and (ii) this finding is consistent with the results of a recent meta-analysis of yield data from mainly perennial fruit crops, which also reported that there is no significant difference in yield between conventional and organic perennial fruit crops [109].

For fruit and vegetables, the evidence for agricultural intensification having resulted in a decline in the nutritional quality has also grown substantially since the publication of the comprehensive systematic literature reviews/meta-analyses of composition differences between organic and conventional cereal crops in 2011 and 2014 [23,24]. Specifically, new evidence from long-term factorial field experiments and retail surveys have increased our understanding of how and to what extent changes in variety choice and agronomic practices affect the concentrations of (i) phenolics, (ii) vitamins, and (iii) toxic metals (e.g., Cd) in fruit and vegetable crops. These studies have also increased our understanding of the large variation between studies and/or crop species found in meta-analyses of comparative composition data [23,24].

The decline in mineral concentrations over time [10,11,12,13] may be explained by changes in crop genetics (e.g., higher yields of modern varieties/hybrids resulting in a dilution effect), and this should be further investigated in the future, for example, by including traditional and modern varieties in factorial field experiments.

Different to wheat and bovine milk, there is still insufficient published data to reliably detect and quantify the effects of intensification for most individual vegetable and fruit species through meta-analyses. Conclusions on the effects of intensification therefore still rely on synthesizing composition data across crop species, using information from studies that compared (i) samples from organic and conventional production or (ii) food composition data published before and after agricultural intensification. However, synthesizing composition data from different crop species does not take into account differences in the consumption of specific fruit and vegetables as part of a MedDiet. It is, therefore, also currently impossible to estimate differences in total intakes of desirable and undesirable/toxic compounds with fruit and vegetables (i) before and after agricultural intensification and (ii) from organic and conventional production.

As with cereals, there is general acceptance that agricultural intensification has resulted in an increase in pesticide residues in fruit and vegetable crops [4,24], and this was confirmed by a recent dietary intervention study which found that (i) increasing fruit and vegetable and wine consumption by changing from a Western diet to a traditional MedDiet with conventional foods will significantly increase urinary pesticide excretion (especially of insecticides; see also Figure 2), while (ii) consuming organic instead of conventional-food-based MedDiets will reduce pesticide exposure by more than 90% [34].

### 3.3. Effects of Intensification on the Nutritional Quality of Meat and Dairy Products

Meat and dairy products are major dietary sources for omega-3 fatty acids (*n*-3), including the very-long-chain omega-3 fatty acids eicosapentaenoic acid (EPA, c5c8c11c14c17 C20:5), docosapentaenoic acid (DPA, c7c10c13c16c19 C22:5), and docosahexaenoic acid (DHA, c4c7c10c13c16c19 C22:6), which were linked to a reduced risk of cardiovascular disease, cancer, and/or obesity, improved body composition, bone density, and foetal development, and enhanced anti-inflammatory, immune, neurological, and cognitive functions [5,6,7,8,9,89,103,104,105,106,107,108,109,110]. Western diets are thought to be deficient in EPA/DPA/DHA, and there are recommendations to significantly increase intakes, especially for pregnant women [6]. Milk and dairy products also contain significant amounts of nutritionally desirable carotenoids, vitamin E, oleic acid, and the mineral micronutrient iodine [5,6,7,8,9,104,105].

The evidence for nutritionally relevant changes in meat and dairy product composition from agricultural intensification comes primarily from studies which compared meat and dairy products produced with contrasting feeding regimes, livestock genotypes, and management systems (organic versus conventional, outdoor versus indoor production). The main changes in livestock production introduced during the period of agricultural intensification over the last 50 years were the (i) increased use of indoor production and concentrate feeds made from cereals and grain legumes (often described as feedlot systems), (ii) reductions in the amount of dry matter intake from grazing/foraging on pasture, (iii) breeding for and/or the use of breeds/hybrids selected for high feed-use efficiency and milk or meat yield/animal from concentrate-feed-based diets, (iii) use of hormones and antibiotics as growth promoters (which is still permitted in North America but not the EU), and (iv) an increased use of veterinary medicine inputs, partially to address negative health impacts resulting from the change to more intensive management practices [5,6,7,8,9,78,111,112,113].

It is now well documented that (i) livestock feeding regimes are the main factor affecting the nutritional and sensory quality of meat and dairy products and (ii) that replacing outdoor-grazing-based high forage intake diets with diets based on concentrate feed made from cereals and grain legumes has a substantial negative effect on the nutritional composition of meat and dairy products, with the largest declines having been reported for concentrations of omega-3 fatty acids (including EPA, DPA, and DHA) [5,6,7,8,9,25,111,112,113].

For example, in beef cattle, traditional forage/grass-based diets resulted in more than three times higher concentrations of total omega-3 and very-long-chain (EPA+DPA+DHA) omega-3 fatty acids in meat of the *longissimus* muscle of bulls from two different cattle breeds (German Holstein and German Simmental bulls), compared with feedlot-type concentrate-based diets [113] (Table 3).

Similarly, the meat of the *longissimus thoracis et lumborum* muscle of Suffolk × ‘Mule’ ram lambs was found to contain more than two times higher total and very-long-chain omega-3 fatty acid concentrations when the animals were finished on forage compared with concentrate-based diets (Table 4) [111].

Differences in omega-3 fatty acid concentrations between pork from pigs reared outdoors with access to pasture and pigs raised indoors on concentrate-only diets were reported to be smaller than those found between meat from grazing-only and concentrate-only ruminant production systems [112]. Specifically, total omega-3, DPA, and DHA concentrations were ~10–20% lower in indoor-reared pigs on concentrate-only diets compared with outdoor-reared pigs on concentrate-based diets with some grazing-based fresh forage intake [112].

Pork from indoor systems also contained significantly lower (~25%) lower α-tocopherol concentrations [112].

A systematic literature review/meta-analysis of composition differences between organic and conventional meat published in 2016 estimated that organic meat has ~50% higher total omega-3 fatty acid concentrations compared with conventional meat when data from all livestock species were pooled [25].

Studies that compared the fatty acid profiles of milk from production systems which used (i) grazing-based, traditional, and (ii) intensified (higher concentrate and/or conserved forage) feeding regimes reported similar trends to those found for ruminant meat in both Europe and the USA; specifically, both total and very-long-chain omega-3 fatty acids decreased significantly with increasing use of concentrate in the dairy diet [5,6,7,8,9,114,115,116,117,118,119]. Also, a systematic literature review/meta-analysis of organic and conventional bovine milk published in 2016 estimated that organic milk has ~60% higher concentrations of very-long-chain omega-3 fatty acids [6].

In dairy cattle, high concentrate diets were also shown to reduce the concentrations of conjugated linoleic acid (CLA), carotenoids, and vitamin E in milk [6,7]. In contrast, Se and I concentrations in bovine milk were found to be significantly lower in production systems with high grazing-based forage intake, compared with high-concentrate diets [6,7]. This is thought to be mainly due to higher concentrate intake because concentrate feeds are routinely fortified with mineral supplements, and concentrate feed use is higher in conventional systems. The lower I levels in organic milk have resulted in concerns that this may lead to I deficiency in countries (e.g., the UK) which do not follow the WHO recommendations to fortify salt with I and where milk/dairy products are the main dietary source for I intake [6].

It is important to note that most Mediterranean small ruminant (meat, goat) production systems have remained grazing-based systems, although (i) the proportion of concentrate and conserved forage in the diet and (ii) the use of improved pastures for grazing and conserved forage production has increased [8,9]. Studies which compared milk from (i) the now widely used semi-intensive (higher concentrate and veterinary medicine inputs) and (ii) traditional forage-based (very low concentrate and veterinary inputs) grazing systems reported smaller differences in omega-3 content, but concentrations were also significantly lower in milk from semi-intensive compared with extensive dairy sheep and goat production systems [Table 5] [8,9].

It is important to note that a range of other practices introduced as part of the intensification of livestock production were also linked to small but significant negative effects on milk quality, and these include the following:

- The use of Swiss Brown dairy cattle genotypes selected for high milk yield from concentrate feed (reduction in omega-3 concentrations in milk) [5];
- The use of pigs with the RN genotype (higher concentrations of total omega-3 fatty acids in pork) [112];
- The robotic milking of dairy cows (reduced concentrations of β-lactoglobulin and total polyunsaturated fatty acid and increased concentrations of the saturated fatty acids lauric acid/C12:0 and myristic acid/C14:0 in milk) [117];
- The use of mineral N fertilizer to increase forage production in grass clover swards used for grazing dairy cattle (reduction in omega-3 fatty acid concentrations in milk) [7].

For dairy cattle, significant interactions between breed and feeding regimes were also reported for both milk yield per cow and the omega-3 concentrations in milk [5]. Compared with traditional Swiss Brown genotypes bred for/selected in alpine outdoor-grazing-based systems (T-SB) in Switzerland, Swiss Brown genotypes that had been selected for high milk yield (HY-SB) from high-concentrate diets in the US produced significantly higher yields on concentrate/conserved-forage-based winter diets but similar yields on grazing-based fresh forage summer diets. In contrast, HY-SB produced milk with significantly lower milk omega-3 fatty acid (ALA, EPA and DPA) concentrations with both winter and summer feeding regimes, although both (i) omega-3 concentrations and (ii) the relative difference between HY-SB and T-SB genotypes was greater with grazing-based summer feeding regimes [5].

Different to crop production, there is, to our knowledge, no evidence that the intensification of livestock nutrition or production systems (organic versus conventional) has increased concentrations of Cd and other toxic metals in meat or dairy products [6,25]. Also, compared to wholegrain cereals, fruit, and vegetables, pesticide residues in meat and dairy products are (i) lower and (ii) more frequently below the limit of detection, and livestock products are therefore not thought to be a major driver for dietary pesticide exposure (Figure 2) [34,74,120,121].

For meat and milk, the evidence for agricultural intensification having resulted in a decline in the nutritional quality has grown substantially since the publication of the comprehensive systematic literature reviews/meta-analyses of composition differences between organic and conventional meat and dairy products [6,25]. Most importantly, the additional evidence described above [8,9,110,111,112,113] confirmed that the use of concentrate feeds (which increased substantially during agricultural intensification) results in substantially lower concentrations of very-long-chain omega-3 fatty (VLC *n*-3) acids EPA, DPA, and DHA in meat and milk from both bovine and small ruminant production.

Since VLC *n*-3 are recognized to be deficient in Western diets, it has been recommended that dietary intakes of VLC omega-3 fatty acids should be doubled, especially during pregnancy [6,25,104,105,110]. Although the consumption of meat is lower than in Western diets, traditional MedDiets are known to result in higher VLC *n*-3 intakes, primarily due to higher fish consumption [50].

Fish, meat, dairy products, eggs and, omega-3 supplements are the only dietary sources for VLC *n*-3, and there is strong evidence that concerns about high concentrations of Cd, the decline in maritime fish stock, and the sustainability of fish farming may limit the ability of increasing VLC *n*-3 intake via fish consumption in the future [6,25,104]. However, the evidence presented in this section suggests that changing to the consumption of meat and dairy products from organic and other extensive outdoor-grazing-based livestock production systems allows for the following:

- A substantial increase in VLC *n*-3 intake as part of MedDiets (e.g., during pregnancy) without an increase in meat or fish consumption;
- The consumers of typical Western diets to follow nutritional advice to substantially reduce meat consumption without reducing their VLC *n*-3 intake or increase fish consumption.

It is important to point out that farm-survey-based studies on bovine milk quality have identified not only (i) *genetics* × *diet interactions* but also (ii) *environment *× *genetics* × *management* and (iii) *genetics* × *agronomy* × *processing* interactions for a range of nutritionally relevant milk composition parameters, including omega-3 PUFA, omega-3/omega-6 ratios, α-tocopherol, and carotenoids [5,6,7,79,114,115,116,117,118,119]. In this context, it is also important to consider that (i) the evidence base for meat from individual livestock species is still relatively small and (ii) a systematic review and meta-analysis of data from studies that compared the nutritional quality of organic and conventional eggs has, to our knowledge, not so far been published. Further research is therefore required to quantify to what extent changing to livestock products from extensive production systems may reverse the negative impacts of the intensification on the nutritional quality of meat, eggs, and dairy products.

### 3.4. Effects of Agricultural Intensification on the Nutritional Quality of Fish

Fish is known to be a good dietary source for omega-3 fatty acids and in particular EPA and DHA, and frequent fish consumption and associated omega-3 fatty acid intake is thought to be a major driver for the health benefits of traditional MedDiets [46,47,48,49,50]. However, in many areas of the Mediterranean and globally, industrial and agricultural pollution has resulted in an increase in the toxic compounds in fish and other seafood. Fish consumption is recognized as a major dietary source for toxic metals such as Cd and Pb [35,122,123] and also a major source of endocrine-disrupting chemicals [122,123,124]. Also, high levels of maternal fish consumption during pregnancy (>3 portions per week) were also linked to rapid child growth and an increased risk of rapid growth in infancy and childhood obesity [124]. This has led to recommendations to limit fish consumption to one portion per week, especially during pregnancy [122,123,124].

Traditionally, MedDiets were based on the consumption of wild fish caught in the Mediterranean Sea and, to a lesser extent, freshwater lakes. However, over the last 60 years, the consumption of farmed fish (i) produced in the Mediterranean (e.g., sea bream, sea brass, dorado) and/or (ii) imported from outside the Mediterranean (e.g., salmon) has increase substantially [125,126,127,128,129,130,131]. Although European consumers prefer wild over farmed fish [126,127] and there is growing concern about the sustainability and environmental impact of fish farming in Europe [128], farmed fish now accounts for more than 50% of fish produced and consumed in Mediterranean countries [125,129,130,131].

Except for salmon (which is produced in Northern Europe and imported by Mediterranean countries), there are, to our knowledge, no studies which compare the nutritional composition of wild and farmed fish [132,133,134,135,136,137]. For salmon, survey-based studies in the US (which compared farmed Atlantic and Pacific wild salmon) and Europe (which compared farmed and wild Atlantic salmon) reported contrasting results for toxic contaminants and omega-3 PUFAs [132,137]. For example, a study in the US reported that farmed Atlantic salmon has higher concentrations of (i) nutritionally desirable omega-3 PUFAs including EPA and DHA but also (ii) nutritionally undesirable toxins such as dioxins, polychlorinated bisphenols, polybrominated diphenol ethers, and pesticides used in fish farming. As a result, the authors recommended that “*young children, women of child-bearing age, pregnant women, and nursing mothers not at significant risk for sudden cardiac death associated with CHD but concerned with health impairments such as reduction in IQ and other cognitive and behavioral effects, can minimize contaminant exposure by choosing the least contaminated wild salmon or by selecting other sources of* (*n*-3) *fatty acids*”. [132]. In contrast, a recent study from Norway [133] found that concentrations of dioxins, dioxin-like PCBs, mercury, and arsenic were three times higher in wild compared to farmed salmon but well below EU-uniform maximum levels for contaminants in food in both farmed and wild fish. The six ICES (International Council for the Exploration of the Sea) PCB concentrations were also higher in wild salmon (5.09 ± 0.83 ng/g) compared with farmed fish (3.34 ± 0.46 ng/g). However, the fat content was substantially (up to three times) higher in farmed salmon, while the proportion of very-long-chain omega-3 PUFAs (EPA and DHA) in fish fat was a substantially (>2 times) higher in wild fish, and the authors concluded that “*Both farmed and wild Atlantic salmon are still valuable sources of eicosapentaenoic acid and docosahexaenoic acid*.” because “*One 150 g portion per week will contribute more (2.1 g and 1.8 g) than the recommended weekly intake for adults*” [133]. However, it should also be noted that the development and introduction of more sustainable feeding regimes (especially a reduction in the use of fish oil and meal) in fish farming is expected to reduce the current levels of very-long-chain omega-3 PUFAs (EPA and DHA) in farmed salmon [134,135].

### 3.5. Limitations of the Currently Available Evidence

Except for wheat, there was insufficient data in all studies which compared food analyses data from before and after agricultural intensification to perform statistically sound comparisons of mineral composition for individual crop species. A study review by Marles [136] also described a concern that the analytical methods used in the 1960s and before differ from those used in the 1980s onwards and that this may have affected the outcome of statistical analyses and/or the estimate of the magnitude of decline in mineral content in food crops. However, they also note that “*The laboratory mineral nutrient analyses of wheat grain varieties and soil samples archived over the last 160 years the Broadbalk Wheat Experiment completed with identical sample preparation and analytical methods, have helped to demonstrate that historical declines in mineral nutrient content of food crops can be real*” [136].

Also, it is not possible to perform reliable comparisons of (i) the phytochemical composition data for crops and (ii) fatty acid and vitamin composition data for meat and dairy products published before and after the period of agricultural intensification due to both a lack of data from before intensification and changes in analytical protocols over time.

Estimates of the effects of intensification therefore rely on recent surveys or experimental studies in which products from modern, intensive production were compared with products from more extensive systems (e.g., organic and traditional extensive farming systems) which do not use many of the agrochemical inputs and breeding innovations (e.g., high-yield selected crop and livestock genotypes) used in intensive systems. However, current extensive systems often use some more recently developed (i) inputs (e.g., biological, botanical, and microbial-fermentation-based pest and disease control products) and (ii) “genetic innovation” (e.g., the majority of organic vegetable, chicken, and to a lesser extent pig production uses modern hybrid genotypes) that were developed for the intensive conventional sector. Since these inputs and innovations were not available before the 1960s, their current use in extensive systems may result in both an over- or underestimate of the effect of intensification.

## 4. Conclusions

There is now substantial evidence that changes in crop and livestock production systems introduced during the period of agricultural intensification had a range of negative impacts on the nutritional composition of crops, meat, and dairy products that form the backbone of the MedDiet.

With respect to compounds linked to health benefits, the evidence for declines in (i) Se, Zn, and phenolic/antioxidant concentrations in cereals, (ii) phenolic/antioxidant concentrations and mineral nutrients in vegetables and/or fruit crops, and (iii) very-long-chain omega-3 fatty acid concentrations in meat and dairy products is of particular concern since insufficient dietary intake of these compounds and associated health problems are known to be widespread globally, and there are recommendations to increase the dietary intake of these compounds [4,6,24,25,26,35,49].

With respect to toxic compounds that were linked to negative health impacts, the increase in dietary pesticide and Cd exposure associated with the rapid expansion of pesticide and P fertilizer use is of particular concern since they were also linked to substantial negative environmental impacts (eutrophication of terrestrial and maritime aquatic ecosystems) and may at least partially explain the high Cd concentrations found in fish and other seafood [34,35].

However, there are only a small number of intervention studies which compared the effect of consuming conventional and organic foods (which are produced without many of the inputs and practices introduced during agricultural intensification) on dietary intakes of nutritionally relevant compounds (see recent reviews of these studies by Vigar et al. [36] and Rempelos et al. [34,35]). However, the available evidence confirmed that organic food consumption can substantially reduce dietary pesticide exposure but found no detectable or only relatively small effects of organic food consumption on dietary intake of minerals and phenolics [34,35,36]. This may indicate that health impacts associated with organic food consumption in observational studies were primarily due an increase in pesticide exposure [34,35,36]. This should be the focus of further research since one recent dietary intervention study also reported that changing from habitual Western diets to a traditional MedDiet not only increases the intake of nutritionally desirable phenolics and minerals but also dietary pesticide exposure [34].

It is important to highlight that changes in nutrition and breeding/selection of both crops and livestock during the period of agricultural intensification were the major and primary drivers for the declines in nutritional quality. Also, there is now some evidence that the overall decline in nutritional quality is due to additive or even synergistic interactions between the changes in crops and animal nutrition and genetics. For example, the use of modern short-straw wheat cultivars together with high N fertilizer inputs resulted in the largest decline in phenolic/antioxidant and mineral micronutrient concentrations in grain. Similarly, the use of Swiss Brown genotypes selected for high milk yield from concentrate feed together with high-concentrate diets resulted in the greatest reduction in omega-3 concentrations in bovine milk.

In many Mediterranean countries, both changes to (i) traditional diets (e.g., higher meat and processed food consumption and lower wholegrain, fruit, and vegetable consumption) and (ii) food composition resulting from agricultural intensification may therefore currently contribute to the increase in major public health problems such as obesity, cardiovascular diseases, metabolic syndrome, and cancer [36,37,38,39,40,41,42,43,44,45,46,47,48,49].

However, it is also important to recognize that some innovations developed and introduced into agricultural practice over the last 60 years had a beneficial effect of the nutritional quality of crops and livestock products. This includes the use of (i) mineral micronutrient fertilizers (e.g., Cu, Se, and Zn) in crop production and (ii) mineral feed supplements (Cu, I, Se, Zn) in livestock production. However, it remains unclear to what extent this has augmented the overall decline in mineral micronutrient concentrations in food crops.

There is now substantial evidence that MedDiets based on organic food consumption may overcome many of the negative nutritional impacts of agricultural intensification. In this context, it is important to consider that there is growing evidence that dietary pesticide intakes resulting from the intensification of conventional food production are a major driver for negative public health impacts.

The following important points should be considered:

- Observational studies have reported that organic food consumption is associated with a lower incidence, and pesticide exposure with a higher incidence, of diseases such as obesity, metabolic syndrome, and cancer (in particular lymphomas) [4,34,35].
- A recent controlled dietary intervention study [34] found that (i) changing from a Western diet with a low conventional fruit and vegetable intake to a MetDiet with a high conventional fruit and vegetable consumption significantly increases dietary pesticide exposure, while (ii) consuming a MedDiet based on organic compared with conventional foods reduces total pesticide exposure by more than 90%.
- A recent observational study from the US [137] reported evidence that increasing dietary pesticide intake with fruit and vegetables reduces the overall positive effect of fruit and vegetable consumption (a major component of MedDiets) on cardiovascular disease (the best-documented positive health impact of MedDiets).

## Figures and Tables

**Table 1:** Percent change in mineral nutrient concentrations in fruit, vegetables, and/or nuts between periods before and after agricultural intensification.

Country [Ref]	Time Periods	Mineral Nutrients Assessed					
Products	Compared	Ca	Mg	Cu	Fe	K	P
UK [10]							
Vegetables	1960s–1990s	−19 *	−45 *	−81 ***	−22 ^T^	−14 ^T^	−6 ^NS^
Fruit	1960s–1990s	0 ^NS^	−11 *	−36 **	−32 **	−20 ***	−1 ^NS^
UK [11]							
Fruit and	1940s–1990s	−6 *	−13 ***	−60 **	−23 ^NS^	−6 ^NS^	+10 ^NS^
Vegetables	1990s–2010s	+3.2 ^NS^	+18 **	+29 *	−35 **	+2 ^NS^	−8 ^NS^
	1940s–2010s	−3 ^T^	−10 **	−49 *	−50 **	−5 ^NS^	+1 ^NS^
UK [13]							
Vegetables	1930s–1980s	−13 ^T^	−21 *	−132 ***	−18 ^T^	−6 ^NS^	+8 ^NS^
Fruits	1930s–1980s	+4 ^NS^	−1 ^NS^	−41 **	−16 *	−11 *	+3 ^NS^
Nuts	1930s–1980s	+9 ^NS^	+6 ^NS^	+8 *	+5 ^NS^	−4 ^NS^	−10 ^NS^
USA [13]							
Vegetables	1930s–2004	−46 ***	+1 ^NS^	−51 ***	−120 ***	−3 ^NS^	−4 ^NS^
Fruits	1930s–2004	−49 ^T^	ND	−44 **	−126 ***	−14 *	−1 ^NS^
Nuts	1930s–2004	ND	ND	+2 ^NS^	−16 ^NS^	ND	ND

*, significant (*p* < 0.05); **, significant (*p* < 0.01); ***, significant (*p* < 0.001); ^NS^, not significant; ND, not determined; ^T^, trend (0.01 > *p* > 0.05).

**Table 2:** Effect of crop protection protocols (organic versus conventional), fertilization (mineral NPK versus farmyard manure), and pre-crop (spring beans versus winter barley) on the concentrations of total phenolics, carotenoids, vitamin C, and vitamin B_9_ in potato tubers, cabbage and lettuce heads, and onion bulbs, and glucosinolates in cabbage heads. Values shown are main effect means ± SE and are expressed on a fresh weight basis; significantly higher means are highlighted in **bold.** Data shown are means ± SE and from Rempelos et al. [27].

Parameter	Crop Protection (P)		Fertilizer Type (F)		Pre-Crop (PC)							
Assessed	CON	ORG	CON	ORG	Spring	Winter	Main Effects			2-Way Interactions		
Crop Species	(CP)	(OP)	(NPK)	(FYM)	Beans	Barley	P	F	PC	P × F	P × PC	F × PC
Phenolics (µg/g)												
Potato ^1^	285	293	277	301	253	259	NS	*	NS	NS	NS	NS
	±14	±14	±13	±14	±10	±8						
Cabbage ^1,2^	11	9	9	12	9	12	T	**	NS	NS	NS	NS
	±1	±1	±1	±1	±1	±1						
Lettuce ^1^	104	109	101	112	89	99	NS	*	T	NS	NS	NS
	±9	±10	±8	±10								
Onion ^1,3^	725	721	740	706	640	641	NS	NS	NS	NS	NS	NS
	±50	±40	±46	±45	±40	±31						
Glucosinolates (µg/g)												
Cabbage ^1^	1374	1233	1128	1479	1432	1670	*	***	NS	NS	NS	NS
	±108	±104	±92	±113	±53	±110						
Carotenoids (µg/g)												
Potato ^1^	0.70	0.77	0.73	0.75	0.78	0.75	T	NS	NS	*	NS	NS
	±0.04	±0.05	±0.04	±0.04	±0.05	±0.04						
Cabbage ^1^	3.5	3.2	3.0	3.7	4.3	4.8	NS	**	NS	T	NS	NS
	±0.4	±0.4	±0.4	±0.4	±0.4	±0.4						
Lettuce ^1^	4.4	5.0	4.5	4.8	4.10	4.64	T	NS	NS	NS	NS	NS
	±0.3	±0.4	±0.4	±0.3	±0.3	±0.3						
Onion ^1^	0.6	0.7	0.7	0.6	0.8	0.7	NS	NS	NS	T	NS	T
	±0.1	±0.1	±0.1	±0.1	±0.1	±0.1						
Vitamin C (µg/g)												
Potato ^1^	95	97	91	101	105	105	NS	***	NS	NS	NS	NS
	±4	±4	±4	±4	±3	±3						
Cabbage ^1^	224	223	211	236	318	337	NS	**	*	NS	NS	NS
	±19	±19	±18	±20	±14	±14						
Lettuce ^1^	7	7	7	7	6	6	NS	NS	NS	NS	NS	NS
	±1	±1	±1	±1	±1	±1						
Onion ^3^	100	93	86	107	115	94	NS	NS	NS	NS	NS	NS
	±21	±13	±12	±22	±26	±15						
Vitamin B_9_ (µg/g)												
Potato ^1^	0.17	0.18	0.17	0.18	0.19	0.19	NS	***	NS	NS	NS	NS
	±0.01	±0.01	±0.01	±0.01	±0.01	±0.01						
Cabbage ^1^	0.44	0.39	0.41	0.43	0.426	0.436	**	T	NS	NS	NS	NS
	±0.02	±0.02	±0.01	±0.02	±0.01	±0.02						
Lettuce ^1^	0.32	0.35	0.32	0.35	0.33	0.33	T	*	NS	NS	NS	NS
	±0.38	±0.02	±0.02	±0.02	±0.03	±0.03						
Onion ^1^	0.42	0.44	0.44	0.42	0.45	0.45	NS	NS	NS	NS	NS	NS
	±0.05	±0.05	±0.05	±0.05	±0.06	±0.05						

CON, conventional; ORG, organic; CP, conventional crop protection protocols used; OP, organic crop protection protocols used; NPK, mineral NPK used as fertilizer; FYM, cattle farmyard manure used as fertilizer; NS, not significant; T, trend (0.1 > *p* > 0.05); *, 0.05 > *p >* 0.01; ** 0.01 > *p* > 0.001; ***, *p* < 0.001; ^1^, data for main effect means and the P × F interaction are from three growing seasons/years (2005, 2006, and 2007), while data for main effects of PC and the P × PC and F × PC interactions are from two growing seasons (2006 and 2007); ^2^, total hydroxycinnamic acid derivatives; ^3^, flavonoids.

**Table 3:** Effect of feeding regime (concentrate versus forage/grass-based diets) and cattle breed (German Holstein versus German Simmental) on individual and total omega-3 (*n*-3) fatty acid and total omega-6 (*n*-6) fatty acid concentrations (% by weight of total fatty acids) and the *n*-6/*n*-3 ratio in the *longissimus* muscle of bulls. Data shown are means ± SE and from Nuernberg et al. [113].

	Cattle Breed				ANOVA Results		
	German Holstein Bulls		German Simmental Bulls		Main Effects		Interaction
Parameter	Concentrate (*n* = 17)	Grass-Based (*n* = 16)	Concentrate (*n* = 16)	Grass-Based (*n* = 15)	Breed	Feed	BreedxFeed
ALA (C18:3*n*-3)	0.34 ± 0.13	1.67 ± 0.13	0.46 ± 0.13	2.22 ± 0.14	*	*	NS
EPA (C20:5*n*-3)	0.14 ± 0.07	0.58 ± 0.08	0.08 ± 0.08	0.94 ± 0.08	*	*	*
DPA (C22:5*n*-3)	0.36 ± 0.09	0.80 ± 0.09	0.29 ± 0.09	1.32 ± 0.10	*	*	*
DHA (C22:6*n*-3)	0.09 ± 0.02	0.15 ± 0.02	0.05 ± 0.02	0.17 ± 0.02	NS	*	NS
Total *n*-3 FA	0.96 ± 0.29	3.25 ± 0.30	0.90 ± 0.30	4.70 ± 0.31	*	*	*
Total *n*-6 FA	6.14 ± 0.82	6.30 ± 0.85	7.73 ± 0.85	9.80 ± 0.15	*	NS	NS
*n*-6/*n*-3 ratio	6.49 ± 0.14	1.94 ± 0.15	8.34 ± 0.15	2.04 ± 0.15	*	*	*

*n*, number of animals assessed; *, significant (*p* < 0.05); NS, not significant; FAs, fatty acids; *n*-3, omega-3; *n*-6, omega-6; ALA, α-linolenic acid; EPA, eicosapentaenoic acid (c5c8c11c14c17 C20:5); DPA, docosapentaenoic acid (c7c10c13c16c19 C22:5); DHA, docosahexaenoic acid (c4c7c10c13c16c19 C22:6).

**Table 4:** Effect of feeding regime (concentrate versus forage/grass-based diets) and sheep breed (pure Welsh Mountain, pure Soay, Suffolk × ‘mule’) on individual and total omega-3 (*n*-3) fatty acid concentrations (% by weight of total fatty acids) in the *longissimus thoracis et lumborum* muscle of ram lambs. Data shown are means and from Fisher et al. [112].

	Sheep Breed			
	Welsh M.	Soay	Suffolk	
Parameter	Grass-Based (*n* = 20)	Grass-Based (*n* = 20)	Concentrate (*n* = 20)	Grass-Based (*n* = 20)
ALA (C18:3*n*-3)	1.6 c	3.3 a	0.7 d	2.3 b
EPA (C20:5*n*-3)	1.0 b	1.8 a	0.4 c	1.3 b
DPA (C22:5*n*-3)	0.9 b	1.6 a	0.8 b	1.5 a
DHA (C22:6*n*-3)	0.3 b	0.7 a	0.3 b	0.6 a
Total *n*-3 FA	3.8	7.4	2.2	5.7

*n*, number of animals assessed, means with the same letter in the same row are not significantly different (*p* < 0.05). FAs, fatty acids; *n*-3, omega-3; ALA, α-linolenic acid; EPA, eicosapentaenoic acid (c5c8c11c14c17 C20:5); DPA, docosapentaenoic acid (c7c10c13c16c19 C22:5); DHA, docosahexaenoic acid (c4c7c10c13c16c19 C22:6).

**Table 5:** Effect of management systems (semi-intensive versus extensive) on concentrations (g/100 g total fatty acids) of individual and total omega-3 (*n*-3) fatty acid in milk fat from mixed dairy sheep/goat flocks in Crete, Greece. Data shown are main effect means ± SE and from Voutzourakis et al. [9].

Parameter	Semi-Intensive (*n* = 10)		Extensive (*n* = 10)
ALA (C18:3*n*-3)	0.55 ± 0.02	***	0.75 ± 0.05
EPA (C20:5*n*-3)	0.046 ± 0.003	***	0.061 ± 0.003
DPA (C22:5*n*-3)	0.075 ± 0.004	***	0.092 ± 0.005
DHA (C22:6*n*-3)	0.019 ± 0.002	ns	0.020 ± 0.002
Total *n*-3 FA	0.97 ± 0.04	***	1.18 ± 0.07

*n*, number of farms from which bulk tank milk samples were collected monthly during the lactation period; ***, *p* < 0.001; ns, *p* > 0.1.

## Data Availability

The datasets generated and/or re-analysed for the results presented in the tables are available from author Leonidas Rempelos on reasonable request.
